# FGF23 as a calciotropic hormone

**DOI:** 10.12688/f1000research.7189.1

**Published:** 2015-12-18

**Authors:** María E. Rodríguez-Ortiz, Mariano Rodríguez

**Affiliations:** 1 Laboratory of Nephrology, IIS-Fundación Jiménez Díaz, Madrid, Spain, 28040, Spain; 2 Nephrology Service and Maimónides Institute for Biomedical Research (IMIBIC), Reina Sofia University Hospital, University of Córdoba, Avda. Menéndez Pidal, S/N, 14004 Córdoba, Spain

**Keywords:** FGF23, calcium, FGF receptor

## Abstract

Maintaining mineral metabolism requires several organs and hormones. Fibroblast growth factor 23 (FGF23) is a phosphatonin produced by bone cells that reduces renal production of calcitriol – 1,25(OH) _2_D _3_ – and induces phosphaturia. The consequences of a reduction in 1,25(OH) _2_D _3_ involve changes in calcium homeostasis. There are several factors that regulate FGF23: phosphorus, vitamin D, and parathyroid hormone (PTH). More recently, several studies have demonstrated that calcium also modulates FGF23 production. In a situation of calcium deficiency, the presence of 1,25(OH) _2_D _3_ is necessary to optimize intestinal absorption of calcium, and FGF23 is decreased to avoid a reduction in 1,25(OH) _2_D _3_ levels.

## Introduction

The regulation of calcium (Ca) and phosphorus (P) is controlled mainly by the parathyroid hormone (PTH), vitamin D, and, to a lesser extent, calcitonin. Fibroblast growth factor 23 (FGF23) was discovered in the early 2000s, initially identified as the cause of several congenital and acquired diseases ^1– 4^. Shimada and collaborators demonstrated the involvement of FGF23 in mineral homeostasis as a regulator of P and vitamin D metabolism ^2, 5^.

FGF23 is a 32 KDa protein produced primarily in bone by osteocytes and osteoblasts. FGF23 acts mainly in the kidney inducing phosphaturia by decreasing the expression of renal cotransporters NaPiIIa and NaPiIIc ^5, 6^. In addition, FGF23 reduces calcitriol – 1,25(OH) _2_D _3_ – by both decreasing 1α-hydroxylase and increasing 24-hydroxylase activities; these enzymes catalyze the synthesis and catabolism of 1,25(OH) _2_D _3_, the active metabolite of vitamin D, respectively ^6^. Parathyroid glands have been shown to be another target for FGF23, as it suppresses both PTH synthesis and secretion ^7^.

In order to exert its biological actions, FGF23 targets a FGF receptor (FGFR). FGF23 belongs to the family of endocrine FGFs, molecules with a very low affinity for their receptors. Thus, the FGFR requires a co-receptor that eases the ligand-receptor binding: αKlotho is the co-receptor for FGF23 ^8^.

In chronic kidney disease (CKD), the accumulation of P stimulates FGF23 production from the early stages of CKD. High levels of FGF23 are thought to contribute to the reduction in renal production of 1,25(OH) _2_D _3_, a key factor in the development of uremic hyperparathyroidism ^9^.

**Figure 1.**
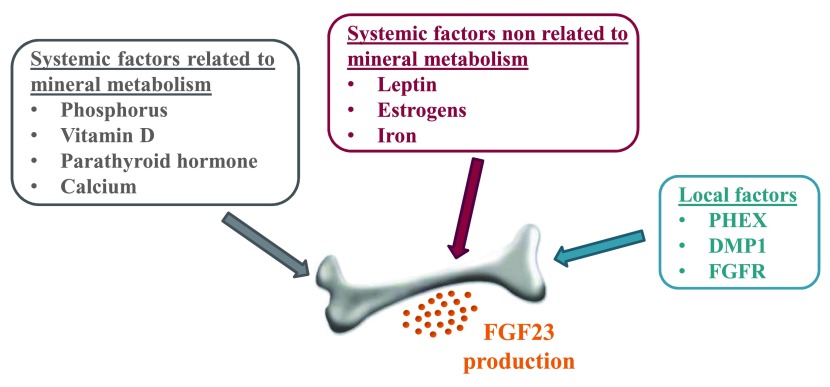
Overview of the main regulators of fibroblast growth factor 23 (FGF23) production.

The regulation of FGF23 production has been investigated by several groups. It is well known that vitamin D upregulates FGF23 *in vivo* and *in vitro* ^10^. PTH also stimulates FGF23 production ^11, 12^. An increase in dietary P stimulates FGF23, although acute increases in serum P fail to increase FGF23 ^9, 13^. PHEX and DMP1, factors involved in the regulation of bone mineralization, also modulate FGF23 expression ^14^. Interestingly, intravenous iron induces transient elevations in the levels of FGF23 ^15^, and recent findings suggest that both leptin and estrogens also influence FGF23 expression ^16, 17^. The main regulators of FGF23 levels are summarized in Figure 1.

Recent studies reveal that the FGF23-Klotho axis is involved in renal ion transport through different mechanisms. FGF23 regulates sodium transport by modulating the sodium-chloride channel (NCC) in distal renal tubules ^18^, and Klotho influences the secretion of potassium by regulating the abundance of the renal outer medullary potassium channel 1 (ROMK1) ^19^.

Over the last few years, experimental and association studies have assessed the role of Ca as a modulator of FGF23 expression. Conversely, FGF23 also modulates renal Ca handling. Both aspects of the association between Ca and FGF23 will be discussed here.

## Modulation of FGF23 expression by calcium

**Figure 2.**
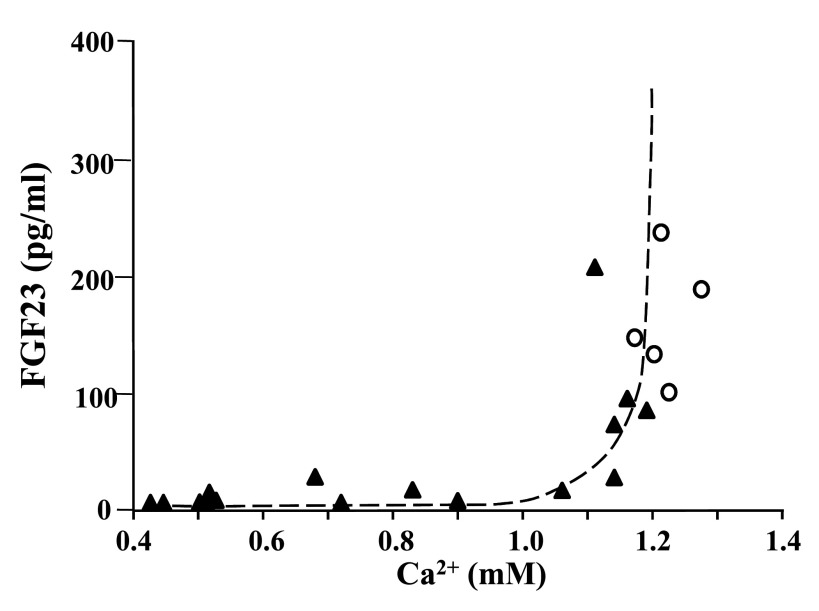
Relationship between serum fibroblast growth factor 23 (FGF23) and ionic calcium in rats fed with a normal diet (r ^2^=0.07; P=0.66) or a calcium and vitamin D-deficient diet (r ^2^=0.73; P<0.001) (dotted line). Adapted from reference 21 (Rodríguez-Ortiz *et al.*, 2012).

Several lines of evidence point out that Ca is a direct regulator of FGF23. In vitamin D receptor (VDR)-null mice, with almost undetectable levels of FGF23, administration of high dietary Ca induced a marked elevation of FGF23 at both mRNA and protein levels ^20^. We obtained similar results using a different experimental approach ^21^. In rats fed with a diet deficient in Ca and vitamin D, the serum levels of FGF23 were extremely low despite very high PTH, which should stimulate FGF23 production. Analyzing the association between FGF23 and Ca ( Figure 2), we found a positive and significant correlation (r ^2^=0.73; P<0.001); interestingly, those rats with serum Ca below 1 mM had reduced FGF23, suggesting a threshold level of Ca above which an increase in FGF23 is allowed. In parathyroidectomized (PTx) rats that presented hypocalcemia, serum FGF23 concentration was low. In these PTx animals, an elevation of serum Ca after an acute (6-hour) infusion of Ca or after the chronic administration of a high-Ca diet produced an increase in serum FGF23. In these experiments, the increase in FGF23 was not explained by an increase in serum concentration of 1,25(OH) _2_D _3_ or P. Gravesen *et al.* ^22^ analyzed the effects of acute changes in serum Ca on FGF23 levels over a shorter period of time. After 60 minutes of hypocalcemic and hypercalcemic clamps, no changes were detected in FGF23, and the acute infusion of Ca in PTx rats did not modify serum FGF23 either. Therefore, it seems that the regulation of FGF23 by Ca is not produced in such a short time.

In recent work by David *et al.* ^23^, the levels of Ca and 1,25(OH) _2_D _3_ were positively associated with FGF23 in wild-type, 1α-hydroxylase ^-/-^ and GCM2 ^-/-^ mice (P=0.048 and P=0.011, respectively) in a multivariate analysis of pooled data. This model is characterized by the absence of 1,25(OH) _2_D _3_ and PTH, and the administration of a rescue diet with a high content of Ca stimulated FGF23 and FGF23 mRNA expression. Importantly, the authors also observed that high Ca stimulated the activity of the FGF23 promoter in osteoblastic cells as well as the levels of FGF23 secreted. The effect of Ca on promoter activity was blocked by the addition of a Ca channel blocker to the culture medium. Conversely, treatment with Ca ionophores increased promoter activity. Although previous work by this group failed to find an effect of Ca on FGF23 promoter activity ^10^, the reason for such apparent disparity of results might reside in the characteristics of the cell lines used and/or the different Ca concentrations used in the experiments.

Studies by Dr Brown’s laboratory examined the role of the calcium-sensing receptor (CaSR) in the regulation of FGF23 by Ca ^24^, as it constitutes the main mechanism of Ca sensing in the tissues involved in mineral homeostasis. They found that the stimulatory effect of Ca on FGF23 was present in the double knockout mice PTH-CaSR. Therefore, they concluded that the CaSR did not mediate the stimulation of FGF23 by Ca in bone cells. In addition, they found that concentrations of 5 mg/dl of P were required to enable the stimulation of FGF23 by Ca. Conversely, regulation of FGF23 by P is abolished when Ca is lower than 8 mg/dl; in fact, they obtained a better correlation between FGF23 and Ca × P (r=0.70) than between FGF23 and Ca or P individually (r=0.65 and 0.58, respectively).

In healthy humans, Vervloet and collaborators analyzed the effect of a Ca and P-rich diet on serum FGF23. Although the effects of the dietary Ca and P were not analyzed separately, the authors found an increase in FGF23 after 36 hours of consumption of the Ca and P-rich diet ^25^. More recently, a cross-sectional study performed in a cohort of middle-aged subjects analyzed the influence of demographic, clinical, and dietary factors on FGF23. In this work, intake of Ca and protein were analyzed individually, with dietary Ca intake significantly associated with higher levels of FGF23 (P=0.01) ^26^. These results are in concordance with previous findings in animals, with higher FGF23 associated with Ca intake.

The effects of acute infusions of sodium citrate and Ca have also been tested in parallel in healthy humans and in uremic patients. No changes in FGF23 were observed after an acute change (120 minutes) in serum Ca concentration in subjects with either normal or reduced renal function ^27^. According to these results, the regulation of FGF23 by Ca does not seem to be as rapid as that of PTH.

Also in the context of CKD, it is worth mentioning that association studies have found significant relationships between FGF23 and Ca. For instance, Imanishi and collaborators reported a positive correlation between FGF23 and Ca (r=0.355, P<0.0001) ^28^. In a prospective study performed in transplant patients, Ca was independently associated with FGF23 levels (P=0.01) ^29^.

Taken together, these data support the idea of Ca as a modulator of FGF23 levels. However, the effect of Ca on FGF23 does not seem to be acute and may not be mediated by CaSR. The meaning of this regulation gains especial relevance in case of hypocalcemia associated with vitamin D deficiency. In such context, the over-suppression of vitamin D by FGF23 would further reduce the calcemia. Therefore, it seems logical that the level of serum Ca conditions that of FGF23. This would therefore constitute a defense mechanism against hypocalcemia.

## Involvement of FGF23 in calcium homeostasis

Despite the fact that FGF23 is considered essentially as a phosphatonin or hormone regulator of P metabolism, there is growing evidence about the involvement of FGF23 in the maintenance of Ca homeostasis.

Three of the four types of FGFR (FGFR1, 3, and 4) can be found in the proximal tubule of the kidney ^6, 30^. The deletion of any of them does not completely block the phosphaturic effect of FGF23, which suggests that this action is not mediated by a single receptor. Gattineni *et al.* identified FGFR1 and 4 as key elements for the phosphaturic response to FGF23 ^31^. While the action of FGF23 increasing phosphaturia takes places upon the interaction with its receptors in proximal tubules of the kidney, FGF23 increases Ca reabsorption by augmenting the expression of the transient receptor potential vanilloid type 5 (TRPV5) in distal tubules. TRPV5 is a glycoprotein essential for the handling of Ca at the kidney level.

It has been reported that Klotho itself controls TRPV5 in an FGF23-independent manner, therefore regulating renal Ca transport ^32^. In addition, Klotho also promotes the trafficking of TRPV5 from inside the epithelial cell ^33^. FGF23 has also been shown to increase Ca reabsorption by regulating the abundance of TRPV5, in an action that is mediated by the signaling pathways ERK1/2, SGK1, and WNK4 ^34^. It might be speculated that the FGF23-independent effects of Klotho appear to be related to the activation and trafficking of the TRPV5, whereas the actions of Klotho acting as a co-receptor of FGFR are involved in the maintenance of P and vitamin D levels. The phenotypical similarities between FGF23 ^-/-^ and Klotho ^-/-^ mutant mice would support this notion.

Kao *et al.* have reported the dysregulatory effect that FGF23 exerts at the cardiovascular level ^35^. FGF23 promotes the phosphorylation of proteins involved in Ca handling in HL-1 atrial cells, such as Ca/calmodulin-dependent protein kinase II (CaMKII) and phospholamban at threonine 17 (PLB). This effect may underlie the relationship between FGF23 and atrial fibrillation described by Seiler and collaborators ^36^.

FGF23 and Klotho contribute to conserving the level of Ca in the organism. This fact might have pathophysiological consequences in the context of CKD. Uremia is characterized by the presence of extraordinarily high levels of FGF23, which may help prevent the Ca loss.

## Conclusion

Systems regulating Ca and P homeostasis are closely related. This is illustrated by FGF23, which regulates Ca and P metabolism and is modulated by both elements. The effect of Ca as a regulator of FGF23 has been widely demonstrated. In studies carried out in experimental animals, Ca deficiency is associated with low FGF23, whereas Ca administration increases its levels. In healthy humans, higher dietary Ca is associated with higher FGF23, although this effect is not observed when Ca is administered acutely. In uremia, some studies point out an association between FGF23 and Ca. On the other hand, very recent work has shown how FGF23 regulates Ca homeostasis, increasing renal reabsorption in a mechanism involving TRPV5. The association between FGF23 and Ca might be relevant in CKD, when there is an imbalance in FGF23 production and risk of unfavorable effects associated with high Ca.

Forthcoming research should be focused on studying in depth the nature of the relationship between FGF23 and Ca, particularly in the context of CKD and its derangements in mineral metabolism. In addition, it is of outstanding importance to unravel the molecular mechanisms and signaling pathways underlying this regulatory feedback loop.

## Abbreviations

Ca, calcium; P, phosphorus; PTH, parathyroid hormone; FGF23, fibroblast growth factor 23; 1,25(OH) _2_D _3_, calcitriol; FGFR, fibroblast growth factor receptor; CKD, chronic kidney disease; PTx, parathyroidectomized; CaSR, calcium-sensing receptor; TRPV5, transient receptor potential vanilloid type 5.

## References

[1] ADHR Consortium: Autosomal dominant hypophosphataemic rickets is associated with mutations in *FGF23*. *Nat Genet.* 2000;26(3):345–8. 10.1038/81664 11062477

[2] Shimada T Mizutani S Muto T: Cloning and characterization of FGF23 as a causative factor of tumor-induced osteomalacia. *Proc Natl Acad Sci U S A.* 2001;98(11):6500–5. 10.1073/pnas.101545198 11344269 PMC33497

[3] Jonsson KB Zahradnik R Larsson T: Fibroblast growth factor 23 in oncogenic osteomalacia and X-linked hypophosphatemia. *N Engl J Med.* 2003;348(17):1656–63. 10.1056/NEJMoa020881 12711740

[4] Larsson T Yu X Davis SI: A novel recessive mutation in fibroblast growth factor-23 causes familial tumoral calcinosis. *J Clin Endocrinol Metab.* 2005;90(4):2424–7. 10.1210/jc.2004-2238 15687325

[5] Shimada T Hasegawa H Yamazaki Y: FGF-23 is a potent regulator of vitamin D metabolism and phosphate homeostasis. *J Bone Miner Res.* 2004;19(3):429–35. 10.1359/JBMR.0301264 15040831

[6] Gattineni J Bates C Twombley K: FGF23 decreases renal NaPi-2a and NaPi-2c expression and induces hypophosphatemia *in vivo* predominantly via FGF receptor 1. *Am J Physiol Renal Physiol.* 2009;297(2):F282–91. 10.1152/ajprenal.90742.2008 19515808 PMC2724258

[7] Ben-Dov IZ Galitzer H Lavi-Moshayoff V: The parathyroid is a target organ for FGF23 in rats. *J Clin Invest.* 2007;117(12):4003–8. 17992255 10.1172/JCI32409 PMC2066196

[8] Urakawa I Yamazaki Y Shimada T: Klotho converts canonical FGF receptor into a specific receptor for FGF23. *Nature.* 2006;444(7120):770–4. 10.1038/nature05315 17086194

[9] Larsson T Nisbeth U Ljunggren O: Circulating concentration of FGF-23 increases as renal function declines in patients with chronic kidney disease, but does not change in response to variation in phosphate intake in healthy volunteers. *Kidney Int.* 2003;64(6):2272–9. 10.1046/j.1523-1755.2003.00328.x 14633152

[10] Liu S Tang W Zhou J: Fibroblast growth factor 23 is a counter-regulatory phosphaturic hormone for vitamin D. *J Am Soc Nephrol.* 2006;17(5):1305–15. 10.1681/ASN.2005111185 16597685

[11] Lavi-Moshayoff V Wasserman G Meir T: PTH increases FGF23 gene expression and mediates the high-FGF23 levels of experimental kidney failure: a bone parathyroid feedback loop. *Am J Physiol Renal Physiol.* 2010;299(4):F882–9. 10.1152/ajprenal.00360.2010 20685823

[12] López I Rodríguez-Ortiz ME Almadén Y: Direct and indirect effects of parathyroid hormone on circulating levels of fibroblast growth factor 23 *in vivo*. *Kidney Int.* 2011;80(5):475–82. 10.1038/ki.2011.107 21525854

[13] Antoniucci DM Yamashita T Portale AA: Dietary phosphorus regulates serum fibroblast growth factor-23 concentrations in healthy men. *J Clin Endocrinol Metab.* 2006;91(8):3144–9. 10.1210/jc.2006-0021 16735491

[14] Martin A Liu S David V: Bone proteins PHEX and DMP1 regulate fibroblastic growth factor *Fgf23* expression in osteocytes through a common pathway involving FGF receptor (FGFR) signaling. *FASEB J.* 2011;25(8):2551–62. 10.1096/fj.10-177816 21507898 PMC3136343

[15] Wolf M Koch TA Bregman DB: Effects of iron deficiency anemia and its treatment on fibroblast growth factor 23 and phosphate homeostasis in women. *J Bone Miner Res.* 2013;28(8):1793–803. 10.1002/jbmr.1923 23505057

[16] Tsuji K Maeda T Kawane T: Leptin stimulates fibroblast growth factor 23 expression in bone and suppresses renal 1alpha,25-dihydroxyvitamin D _3_ synthesis in leptin-deficient mice. *J Bone Miner Res.* 2010;25(8):1711–23. 10.1002/jbmr.65 20200981

[17] Carrillo-López N Román-García P Rodríguez-Rebollar A: Indirect regulation of PTH by estrogens may require FGF23. *J Am Soc Nephrol.* 2009;20(9):2009–17. 10.1681/ASN.2008121258 19628670 PMC2736770

[18] Andrukhova O Slavic S Smorodchenko A: FGF23 regulates renal sodium handling and blood pressure. *EMBO Mol Med.* 2014;6(6):744–59. 10.1002/emmm.201303716 24797667 PMC4203353

[19] Cha SK Hu MC Kurosu H: Regulation of renal outer medullary potassium channel and renal K ^+^ excretion by Klotho. *Mol Pharmacol.* 2009;76(1):38–46. 10.1124/mol.109.055780 19349416 PMC2701452

[20] Shimada T Yamazaki Y Takahashi M: Vitamin D receptor-independent FGF23 actions in regulating phosphate and vitamin D metabolism. *Am J Physiol Renal Physiol.* 2005;289(5):F1088–95. 10.1152/ajprenal.00474.2004 15998839

[21] Rodriguez-Ortiz ME Lopez I Muñoz-Castañeda JR: Calcium deficiency reduces circulating levels of FGF23. *J Am Soc Nephrol.* 2012;23(7):1190–7. 10.1681/ASN.2011101006 22581996 PMC3380648

[22] Gravesen E Mace ML Hofman-Bang J: Circulating FGF23 levels in response to acute changes in plasma Ca ^2+^. *Calcif Tissue Int.* 2014;95(1):46–53. 10.1007/s00223-014-9861-8 24801007

[23] David V Dai B Martin A: Calcium regulates FGF-23 expression in bone. *Endocrinology.* 2013;154(12):4469–82. 10.1210/en.2013-1627 24140714 PMC3836077

[24] Quinn SJ Thomsen AR Pang JL: Interactions between calcium and phosphorus in the regulation of the production of fibroblast growth factor 23 *in vivo*. *Am J Physiol Endocrinol Metab.* 2013;304(3):E310–20. 10.1152/ajpendo.00460.2012 23233539 PMC3566433

[25] Vervloet MG van Ittersum FJ Büttler RM: Effects of dietary phosphate and calcium intake on fibroblast growth factor-23. *Clin J Am Soc Nephrol.* 2011;6(2):383–9. 10.2215/CJN.04730510 21030580 PMC3052230

[26] di Giuseppe R Kühn T Hirche F: Potential Predictors of Plasma Fibroblast Growth Factor 23 Concentrations: Cross-Sectional Analysis in the EPIC-Germany Study. *PLoS One.* 2015;10(7):e0133580. 10.1371/journal.pone.0133580 26193703 PMC4508099

[27] Wesseling-Perry K Wang H Elashoff R: Lack of FGF23 response to acute changes in serum calcium and PTH in humans. *J Clin Endocrinol Metab.* 2014;99(10):E1951–6. 10.1210/jc.2014-2125 25062462 PMC5393489

[28] Imanishi Y Inaba M Nakatsuka K: FGF-23 in patients with end-stage renal disease on hemodialysis. *Kidney Int.* 2004;65(5):1943–6. 10.1111/j.1523-1755.2004.00604.x 15086938

[29] Evenepoel P Naesens M Claes K: Tertiary 'hyperphosphatoninism' accentuates hypophosphatemia and suppresses calcitriol levels in renal transplant recipients. *Am J Transplant.* 2007;7(5):1193–200. 10.1111/j.1600-6143.2007.01753.x 17359508

[30] Andrukhova O Zeitz U Goetz R: FGF23 acts directly on renal proximal tubules to induce phosphaturia through activation of the ERK1/2-SGK1 signaling pathway. *Bone.* 2012;51(3):621–8. 10.1016/j.bone.2012.05.015 22647968 PMC3419258

[31] Gattineni J Alphonse P Zhang Q: Regulation of renal phosphate transport by FGF23 is mediated by FGFR1 and FGFR4. *Am J Physiol Renal Physiol.* 2014;306(3):F351–8. 10.1152/ajprenal.00232.2013 24259513 PMC3920047

[32] Chang Q Hoefs S van der Kemp AW: The beta-glucuronidase klotho hydrolyzes and activates the TRPV5 channel. *Science.* 2005;310(5747):490–3. 10.1126/science.1114245 16239475

[33] Wolf MT An SW Nie M: Klotho up-regulates renal calcium channel transient receptor potential vanilloid 5 (TRPV5) by intra- and extracellular *N*-glycosylation-dependent mechanisms. *J Biol Chem.* 2014;289(52):35849–57. 10.1074/jbc.M114.616649 25378396 PMC4276853

[34] Andrukhova O Smorodchenko A Egerbacher M: FGF23 promotes renal calcium reabsorption through the TRPV5 channel. *EMBO J.* 2014;33(3):229–46. 10.1002/embj.201284188 24434184 PMC3983685

[35] Kao YH Chen YC Lin YK: FGF-23 dysregulates calcium homeostasis and electrophysiological properties in HL-1 atrial cells. *Eur J Clin Invest.* 2014;44(8):795–801. 10.1111/eci.12296 24942561

[36] Seiler S Cremers B Rebling NM: The phosphatonin fibroblast growth factor 23 links calcium-phosphate metabolism with left-ventricular dysfunction and atrial fibrillation. *Eur Heart J.* 2011;32(21):2688–96. 10.1093/eurheartj/ehr215 21733911

