# Supplementary material for: FGF23 as a calciotropic hormone
Source: F1000Res. 2015 Dec 18;4:F1000 Faculty Rev-1472. [Version 1] doi: 10.12688/f1000research.7189.1 (PMC4815615; doi:10.12688/f1000research.7189.1)
Supplement: Peer review history

# Peer review: Referee response for version 1

**DOI:** 10.5256/f1000research.7745.r11651

**Published:** 2015-12-18

**Authors:** Jordi Bover

**Affiliations:** 1 Department of Nephrology, Puigvert Foundation , Barcelona, Spain

This paper not only provides a top-of-art review on the issue but it is also of outstanding quality, in correspondence with the reputation of the authors.

I have read this submission. I believe that I have an appropriate level of expertise to confirm that it is of an acceptable scientific standard.

# Peer review: Referee response for version 1

**DOI:** 10.5256/f1000research.7745.r11650

**Published:** 2015-12-18

**Authors:** Reinold G Erben

**Affiliations:** 1 Unit of Physiology, Pathophysiology, and Experimental Endocrinology, University of Veterinary Medicine Vienna, Vienna, Austria

I have read this submission. I believe that I have an appropriate level of expertise to confirm that it is of an acceptable scientific standard.
